# Background data for modulus mapping high-performance polyethylene fiber morphologies

**DOI:** 10.1016/j.dib.2016.11.071

**Published:** 2016-11-24

**Authors:** Kenneth E. Strawhecker, Emil J. Sandoz-Rosado, Taylor A. Stockdale, Eric D. Laird

**Affiliations:** U.S. Army Research Laboratory, RDRL-WMM-G, Aberdeen Proving Ground, MD 21005-5069, USA

**Keywords:** Atomic force microscopy, AMFM, Ultra-high-molecular-weight Polyethylene, UHMWPE, Modulus mapping

## Abstract

The data included here provides a basis for understanding “Interior morphology of high-performance polyethylene fibers revealed by modulus mapping” (K.E. Strawhecker, E.J. Sandoz-Rosado, T.A. Stockdale, E.D. Laird, 2016) [1], in specific: the multi-frequency (AMFM) atomic force microscopy technique and its application to ultra-high-molecular-weight Polyethylene (UHMWPE) fibers. Furthermore, the data suggests why the Hertzian contact mechanics model can be used within the framework of AMFM theory, simple harmonic oscillator theory, and contact mechanics. The framework is first laid out followed by data showing cantilever dynamics, force-distance spectra in AC mode, and force-distance in contact mode using Polystyrene reference and UHMWPE. Finally topography and frequency shift (stiffness) maps are presented to show the cases where elastic versus plastic deformation may have occurred.

**Specifications Table**

**Table:** 

Subject area	*Physics & Physical Chemistry*
More specific subject area	*Atomic Force Microscopy (AFM) & Ultra-high-molecular-weight Polyethylene (UHMWPE)*
Type of data	*Figure*
How data was acquired	*Data collected using the Cypher AFM*
Data format	*Raw*
Experimental factors	*Spin-cast Polystyrene reference, interior-exposed UHMWPE fiber*
Experimental features	*Data elucidates the AMFM multi-frequency technique and its application to UHMWPE fiber samples*
Data source location	*Aberdeen Proving Ground, MD, USA*
Data accessibility	*Data is with this article*

**Value of the data**

• This data shows the applicability of the multi-frequency (AMFM) modulus mapping AFM technique to UHMWPE fiber samples.
• The data provides a basis for understanding the interaction of an AFM tip with the material to recover tip-sample stiffness.
• The data helps to provide an estimate of the peak force experienced through the tip-sample interaction as well as provides a reason for applying a Hertzian analysis to the AFM data.

## 1 Data

In order to establish the utility of the multi-frequency technique for the purpose of this study [1], details are provided here to include resonant frequency and thermal tune spectra, AC and contact mode force curves. Additionally, Hertzian contact mechanics model fits are applied to curves performed on both PS and UHMWPE. Finally, topography and frequency (i.e. stiffness) maps are shown from before and after the force curve experiments.

## 2 Experimental design, materials and methods

### 2.1 Atomic force microscopy, AMFM theory

**Fig. 1:**
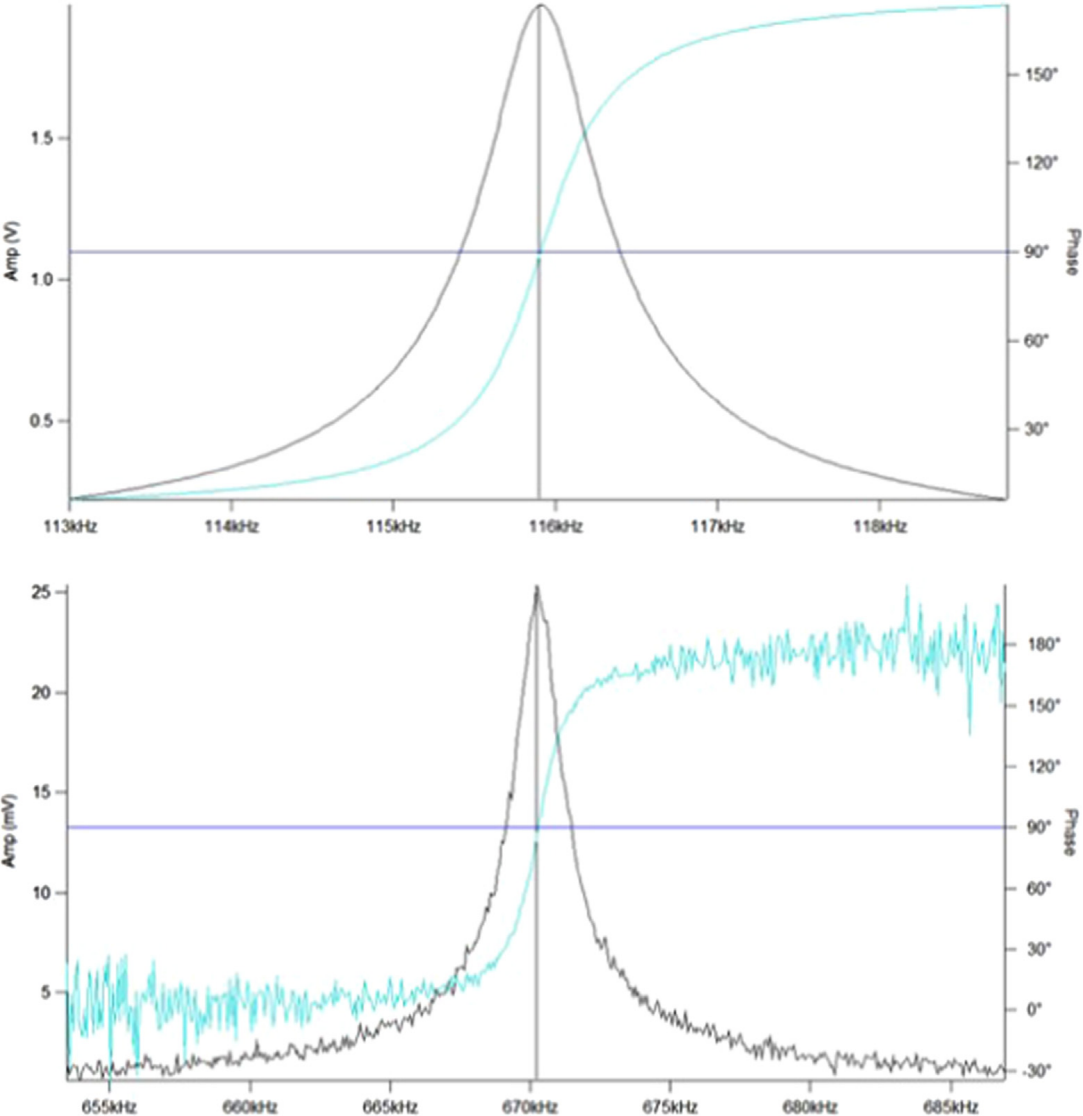
First (top) and second (bottom) bending mode resonant frequency spectra.

**Fig. 2:**
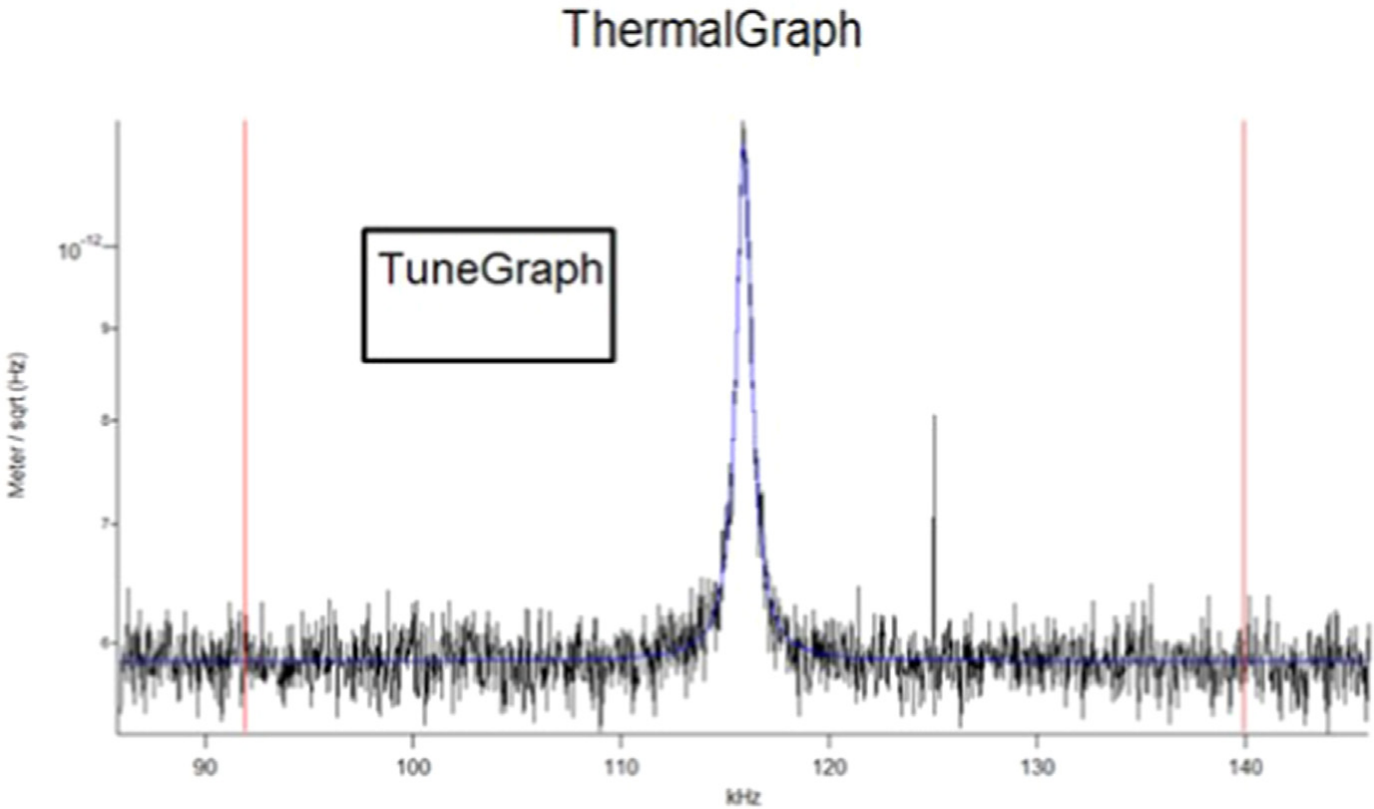
Typical thermal tune plot to be used in the equipartition theorem to calculate the spring constant. Here the spring constant was 4.32 nN/nm.

AMFM is a multi-frequency technique where the cantilever is excited at its mode 1 and mode 2 bending frequencies simultaneously. Background as to how the mode works as is appied using a contact mechanics model are found in the literature [2]. While this AFM technique has similarities to other AFM modulus mapping techniques such as fast force curves, although these other techniques include different cantilever and contact dynamics as well as typically a different contact mechanics model. The first bending mode is used in the feedback loop for standard AFM tapping imaging. The mode 2 bending frequency is used to evaluate the tip-sample stiffness and solve for the contact modulus through the following calculation. These resonant frequency spectra are shown in Fig. 1. The spring constant is measured by the thermal tune method [3]. Data illustrating this is seen in Fig. 2.

**Fig. 3:**
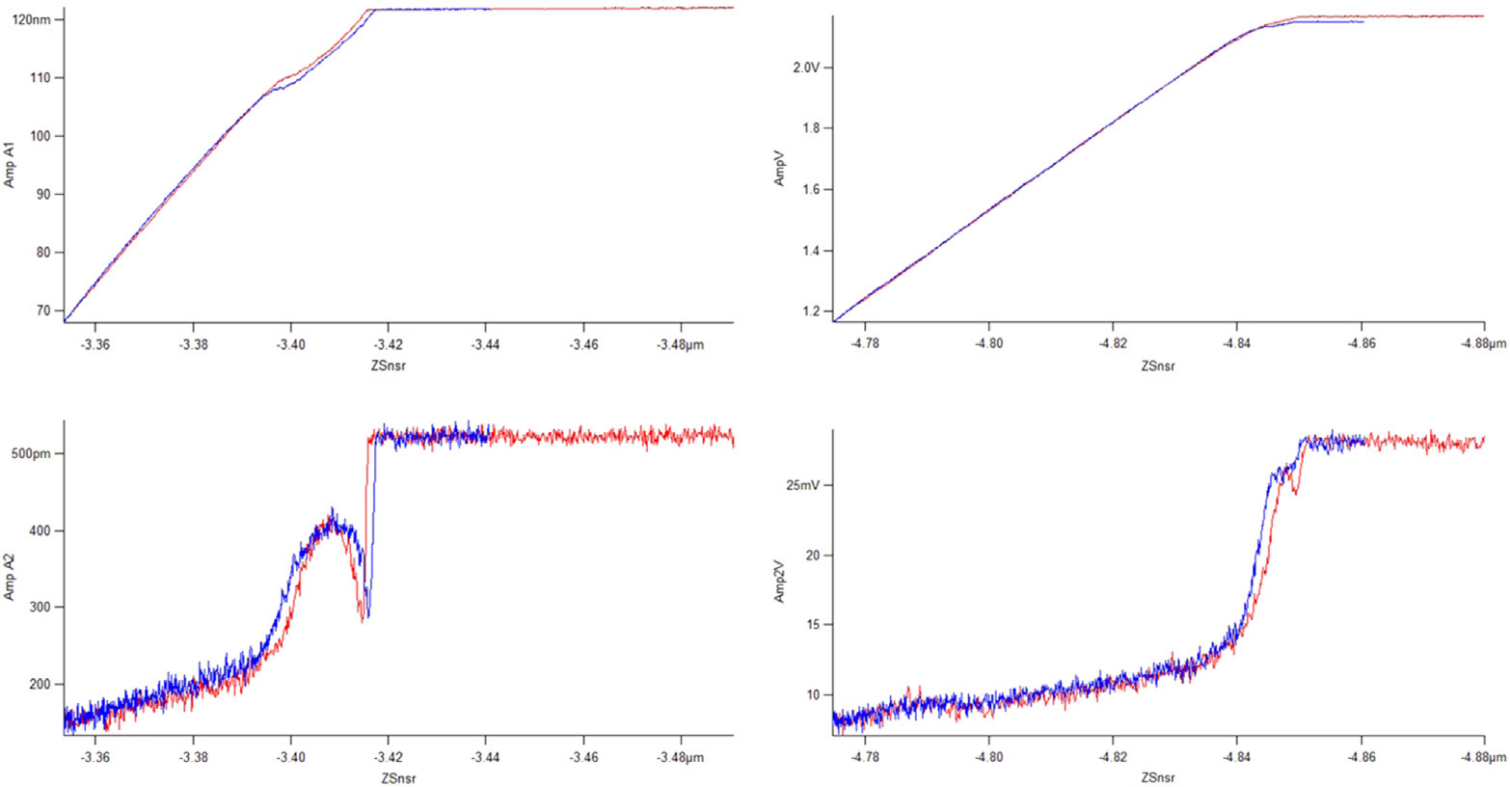
Amplitude versus distance for the first (top) and second (bottom) bending modes. On PS at the left and UHMWPE at the right.

**Fig. 4:**
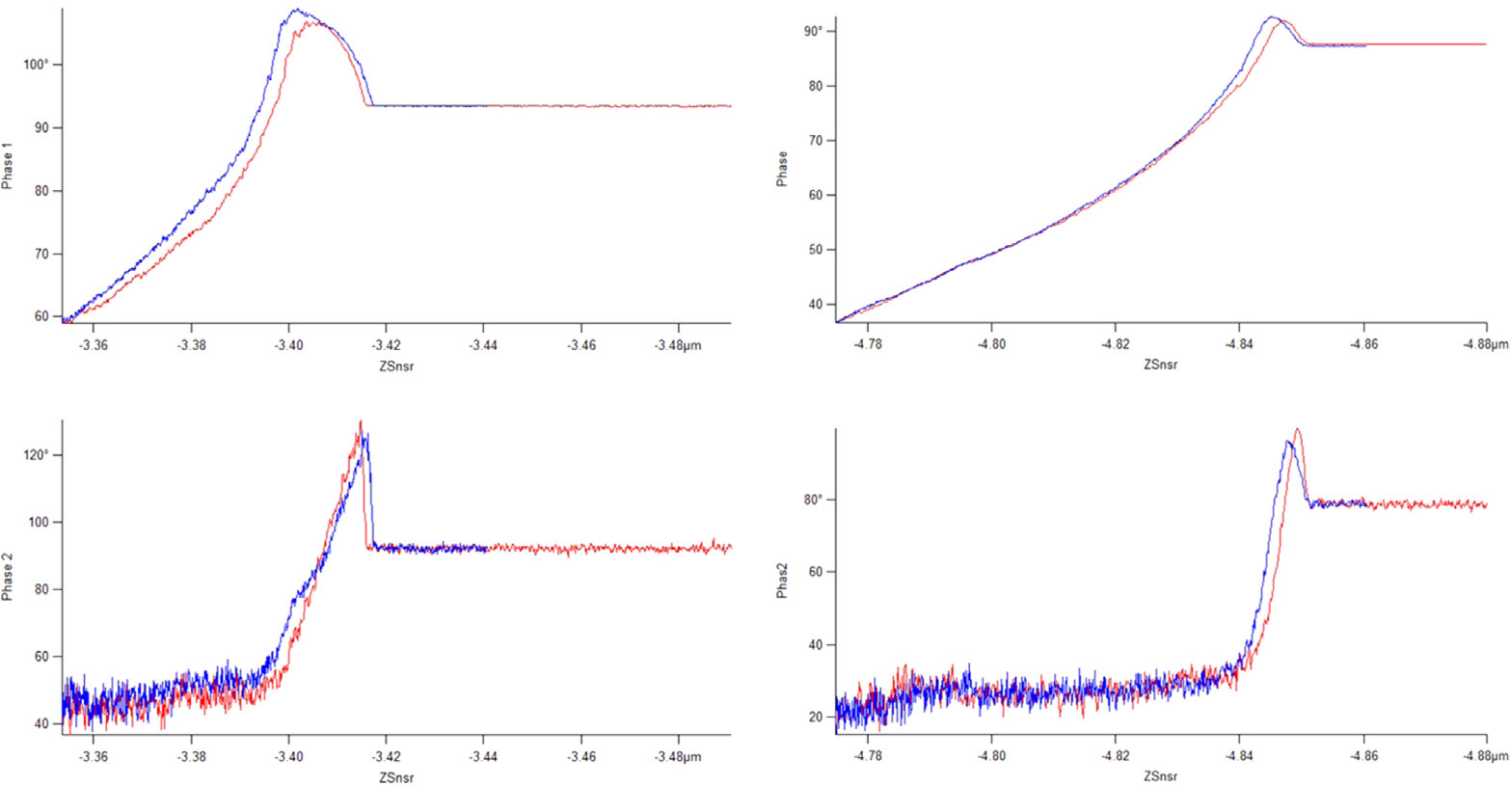
Phase versus distance for the first (top) and second (bottom) bending modes. On PS at the left and UHMWPE at the right.

To illustrate the cantilever dynamics, Fig. 3, Fig. 4 show the amplitude versus distance and the phase versus distance spectra, respectively. These are shown for both the spin-cast PS (Bruker) and the UHMWPE (prepared using a focused-ion-beam notching technique) [4]. What should be noted from these plots is the region of operation for the multi-frequency technique, in specific, the bottom region of the first mode amplitude plot is the imaging amplitude setpoint (1 V). This is far from the upper region which shows hysteresis and attractive features (phase greater than 90 degrees). Additionally, the second mode amplitude is kept constant at 25 mV which is the top region of the amplitude versus distance (Fig. 3, bottom plots). The phase shift in this region (Fig. 4, bottom plots) is relevant because it indicates the frequency shift which occurs, the measureable which is proportional to the tip-sample stiffness. According to reference [5], operating with such aggressive setpoints in the first bending mode (50% reduction from the free-air oscillation amplitude) causes the repulsive forces to dominate the second bending mode measurement (frequency shift).

**Fig. 5:**
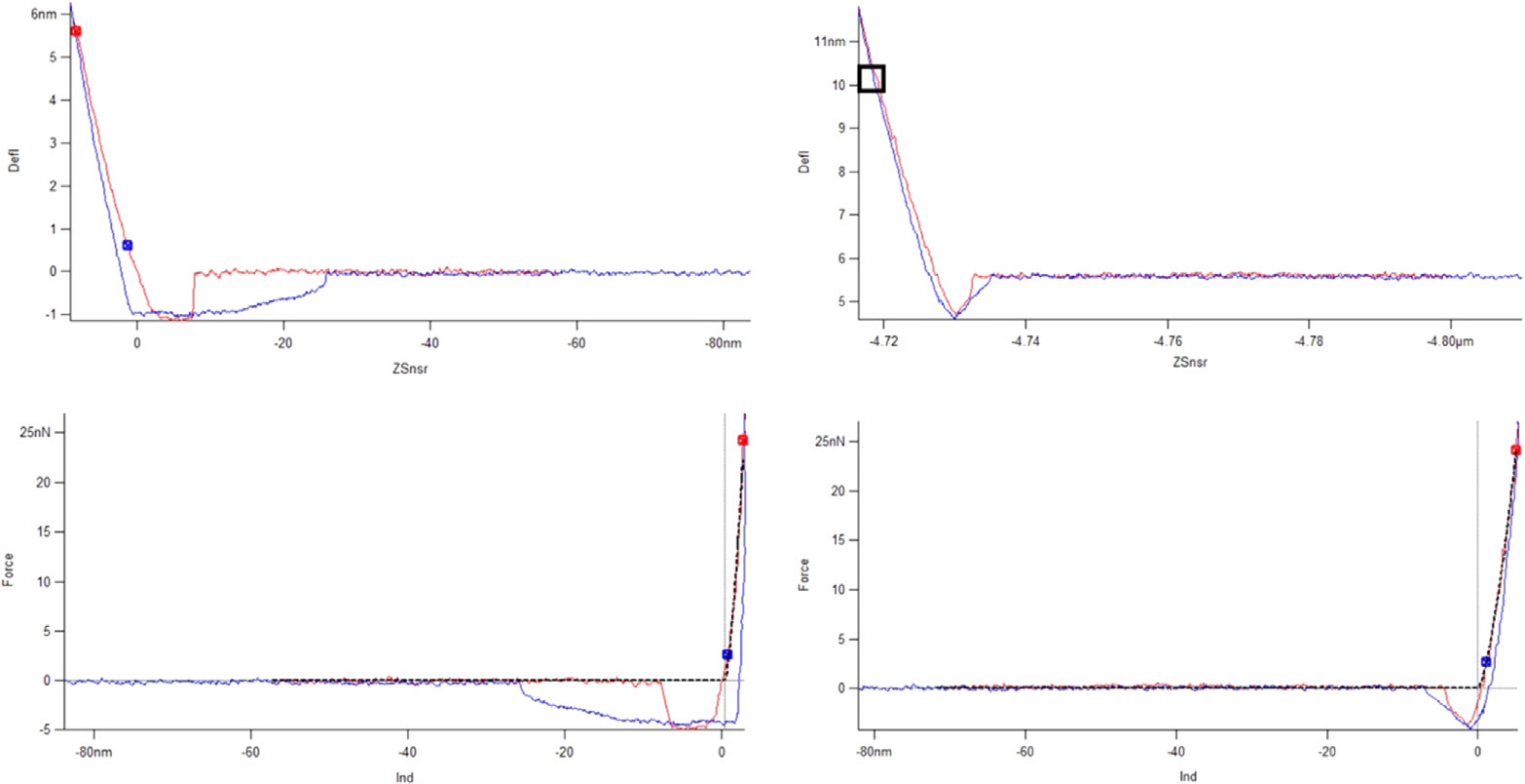
Contact mode deflection versus distance (top) and force versus indentation at low force trigger for PS (left) and UHMWPE (right). Dotted line fit to a Hertzian model on the force versus indentation plots.

**Fig. 6:**
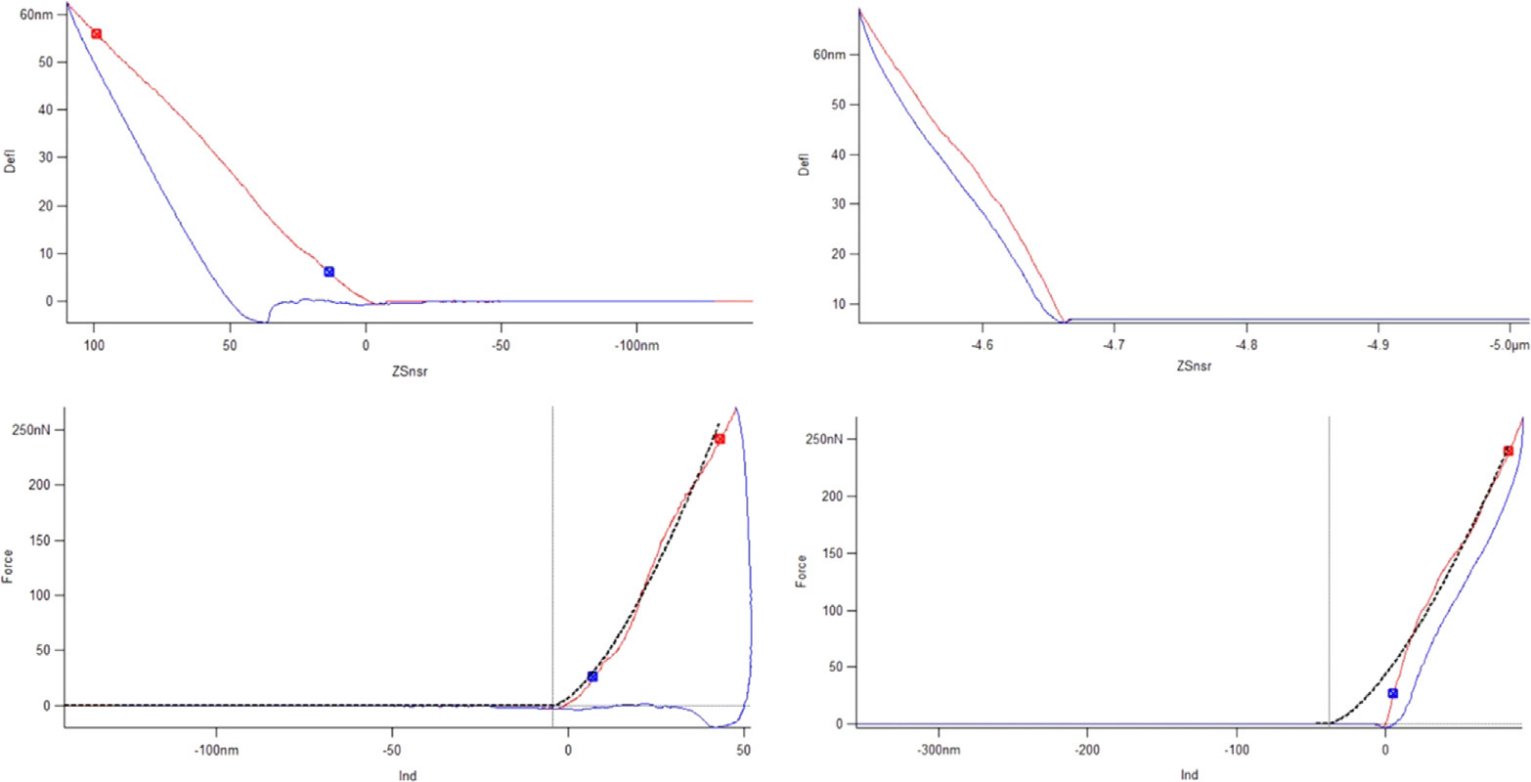
Contact mode deflection versus distance (top) and force versus indentation at high force trigger for PS (left) and UHMWPE (right). Dotted line fit to a Hertzian model on the force versus indentation plots.

Next, force versus distance curves were made on PS and UHMWPE to low deflection triggers (approximately 6 nm deflection, or 25 nN) and these are shown both as force-distance and force-indentation plots in Fig. 5. These were performed at a rate of 1 Hz. On the other hand, the fast interaction of the tip with the sample in AMFM modulus mapping imaging occurs in such a way that time-averaged signals must be used. Additionally, it should be noted that the second mode resonance is driven such that the amplitude is on the order of 0.5 nm, as seen in Fig. 3 bottom, where the full amplitude is marked as 500 picometers. (Through proper calibration of the optical lever sensitivity for the second bending mode, this amplitude was found to be 20% larger [5].). An amplitude of this magnitude is a small fraction of the low trigger force curve shown in Fig. 5. A box is shown on the upper right force-distance curve and the lower right force-indentation curve in Fig. 5 to show the region where the second bending mode oscillation is expected to dominate the AMFM stiffness measurement. This region itself corresponds to a peak force on the order of 1–5 nN and indentation depth on the order of 1 nm, the overall peak force would then be on the order of 10–50 nN since the first bending mode interaction (estimated to be 10–50 nN) would need to be added. From this it can be seen that the main assumptions of the Hertzian analysis are satisfied: that attractive forces are not being probed and that the contact is elastic. In Fig. 5, and also in Fig. 6 where a much larger force trigger is used, the dotted lines along the force versus indentation curves correspond to a Hertzian model applied to these force curves. Here, the spherical tip shape assumed yielded a radius of 2.6 nm to give a modulus of 2.7 GPa on the PS sample. This same radius returned a value of 0.7 GPa for UHMWPE. On the other hand, the appropriate spherical tip radius used in the AMFM modulus maps was 10 nm (modulus of 2.7 GPa on PS and again 0.7 GPa on UHMWPE). In this assessment using standard force curves, the application of the Hertzian model to these materials may not be optimal and another model may be more appropriate. Nonetheless, while a different tip radius is used in the force curve model (versus the AMFM model), both standard force curves and AMFM modulus mapping appear to return the same value for the UHMWPE modulus in this brief study.

**Fig. 7:**
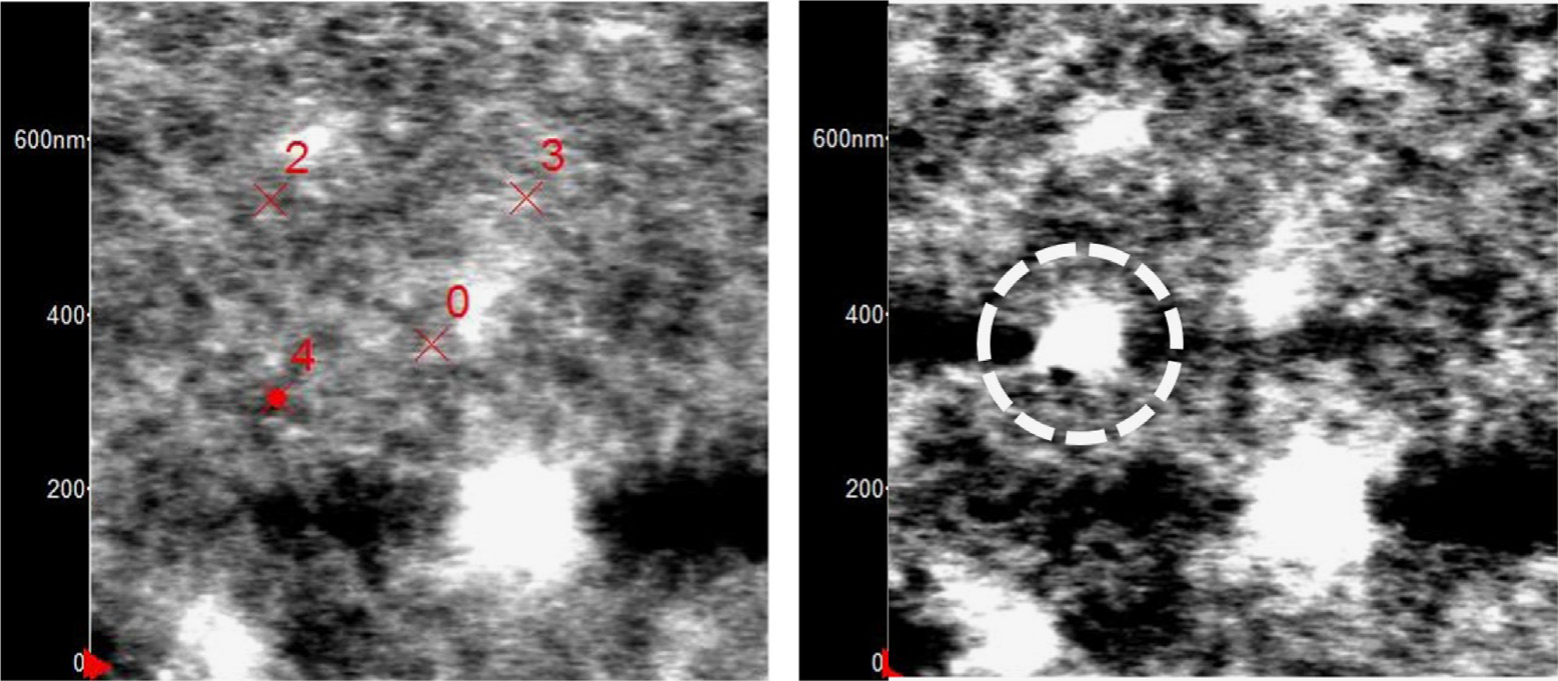
PS topography before force curves (left) and after (right). AC curves from Fig. 3, Fig. 4 were taken at spot 2, low trigger from Fig. 5 at spot 3, and high trigger from Fig. 6 at spot 4. Note the hole and pileup in the PS at right corresponding to the position of spot 4.

**Fig. 8:**
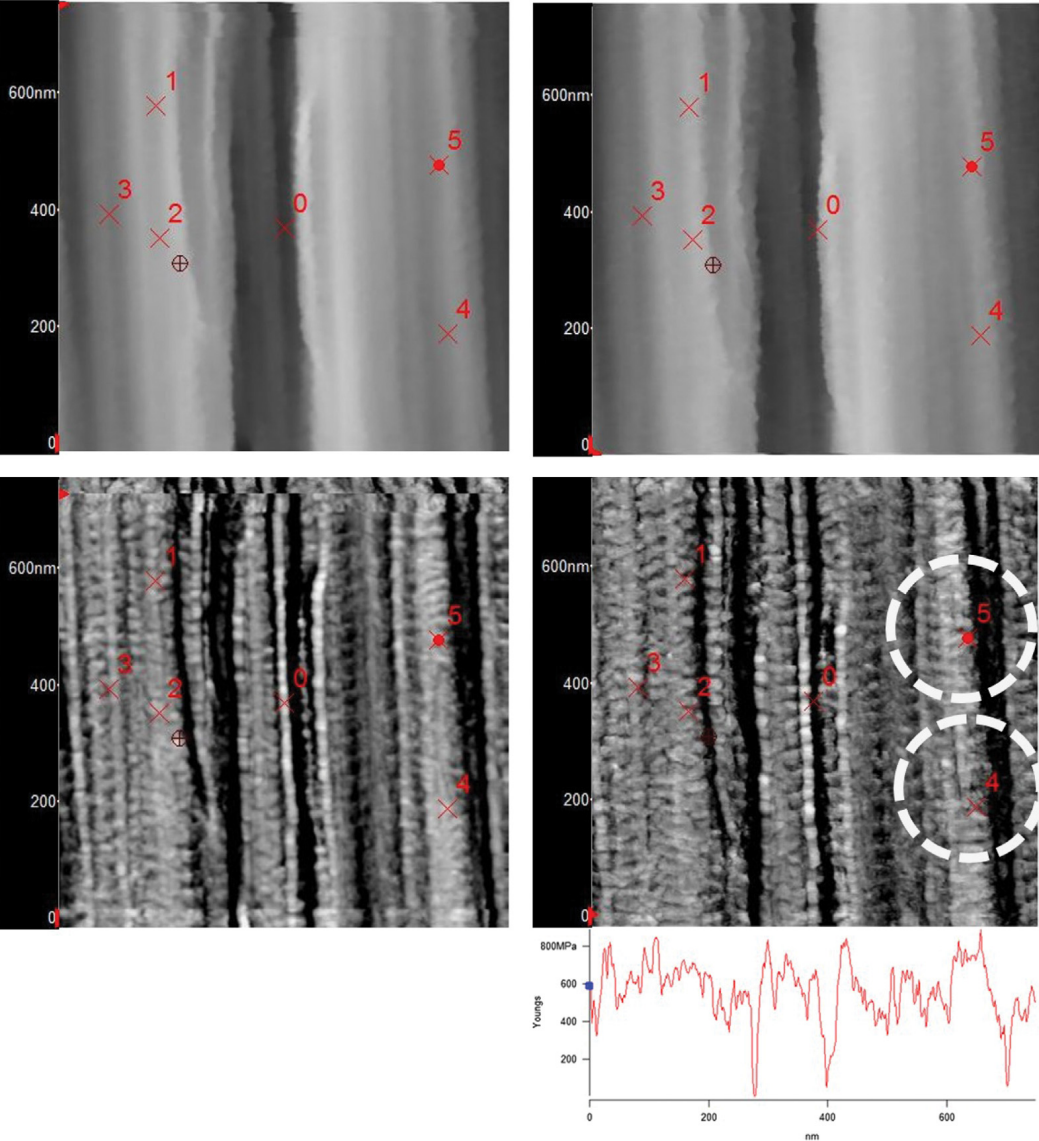
UHMWPE topography (top) before force curves (left) and after (right). Corresponding AMFM modulus maps are directly below. AC curves from Fig. 3, Fig. 4 were taken at spot 2, low trigger from Fig. 5 at spot 3, and high trigger from Fig. 6 at spot 4 and repeated at spot 5. No hole is visible in the topography but darkened region appears in the modulus map indicating a possible mechanical change to the structure of the UHMWPE. At bottom right is a modulus profile from the last line scan in the map immediately above it showing the modulus values to range from 0–800 MPa.

To understand better the limitation of force curve analysis, the trigger threshold was increased to 250 nN (10 times greater) and the results from force-distance and force-indentation for PS and UHMWPE are shown in Fig. 6. Interestingly, the PS indicated much larger plastic indentation characteristics while the UHMWPE appeared to be more elastic. The PS topography images before and after are shown in Fig. 7 and indicate a hole and pile-up associated with the high trigger force indent. On the other hand, the UHMWPE topography images before and after, shown in Fig. 8 (top images) do not show a detectable indent. However, the frequency shift maps (bottom images, Fig. 8) do show two small low stiffness (dark, negative frequency shift) regions at the position where the high trigger force curves were collected. This indicates a rubbery elastic behavior where the UHMWPE is able to recover very quickly, on the order of the time of the force curves (1 s) but it also indicates that in some way the morphology is mechanically different, at least at the immediate time after when it was imaged by AMFM modulus mapping. The calculated modulus values using the Hertzian analysis with a ~10 nm spherical tip radius are shown for a single line scan (the last line) of the bottom-right frequency map in Fig. 8. The values range from 0–800 MPa.
